# Functional Electrical Stimulation for Foot Drop in Post-Stroke People: Quantitative Effects on Step-to-Step Symmetry of Gait Using a Wearable Inertial Sensor

**DOI:** 10.3390/s21030921

**Published:** 2021-01-29

**Authors:** Giulia Schifino, Veronica Cimolin, Massimiliano Pau, Maira Jaqueline da Cunha, Bruno Leban, Micaela Porta, Manuela Galli, Aline Souza Pagnussat

**Affiliations:** 1 Rehabilitation Sciences Graduate Program, Universidade Federal de Ciências da Saúde de Porto Alegre (UFCSPA), Porto Alegre 90050-170, Brazil; giuliaschifino@hotmail.com (G.S.); maira.estudo@gmail.com (M.J.d.C.); alinespagnussat@gmail.com (A.S.P.); 2 Movement Analysis and Rehabilitation Laboratory, Universidade Federal de Ciências da Saúde de Porto Alegre (UFCSPA), Porto Alegre 90050-170, Brazil; 3 Department of Electronics, Information and Bioengineering, Politecnico di Milano, Piazza Leonardo da Vinci 32, 20133 Milano, Italy; manuela.galli@polimi.it; 4 Department of Mechanical, Chemical and Materials Engineering, University of Cagliari, Piazza d’Armi, 09123 Cagliari, Italy; massimiliano.pau@dimcm.unica.it (M.P.); bruno.leban@dimcm.unica.it (B.L.); m.porta@dimcm.unica.it (M.P.); 5 Department of Physiotherapy, Universidade Federal de Ciências da Saúde de Porto Alegre (UFCSPA), Porto Alegre 900050-170, Brazil

**Keywords:** foot drop stimulation, gait, symmetry, stroke, inertial measurement sensor

## Abstract

The main purpose of the present study was to assess the effects of foot drop stimulators (FDS) in individuals with stroke by means of spatio-temporal and step-to-step symmetry, harmonic ratio (HR), parameters obtained from trunk accelerations acquired using a wearable inertial sensor. Thirty-two patients (age: 56.84 ± 9.10 years; 68.8% male) underwent an instrumental gait analysis, performed using a wearable inertial sensor before and a day after the 10-session treatment (PRE and POST sessions). The treatment consisted of 10 sessions of 20 min of walking on a treadmill while using the FDS device. The spatio-temporal parameters and the HR in the anteroposterior (AP), vertical (V), and mediolateral (ML) directions were computed from trunk acceleration data. The results showed that time had a significant effect on the spatio-temporal parameters; in particular, a significant increase in gait speed was detected. Regarding the HRs, the HR in the ML direction was found to have significantly increased (+20%), while those in the AP and V directions decreased (approximately 13%). Even if further studies are necessary, from these results, the HR seems to provide additional information on gait patterns with respect to the traditional spatio-temporal parameters, advancing the assessment of the effects of FDS devices in stroke patients.

## 1. Introduction

Stroke and cerebrovascular disease are leading causes of morbidity, mortality, and disability and represent the most common reason for long-term care not only in developed countries, but also in low- and middle-income countries where stroke is the fourth-leading cause of disability among people older than age 65 [1]. Stroke may severely affect a wide range of motor skills at different levels, including upper and lower limb functioning, particularly due to muscular weakness or partial paralysis (often restricted to one side of the body), which are present in more than 80% of individuals [2]. Hemiparetic individuals often suffer from limitations in mobility and the most common post-stroke impairment that affects gait is foot drop [3]. This motor impairment is associated with the weakness or lack of voluntary control in ankle dorsiflexors and/or the increased spasticity of plantar flexor muscles [4,5,6]. Foot drop interferes with ankle dorsiflexion during the swing phase of gait and contributes to the disruption in weight acceptance and weight transfer in the initial foot contact and stance phases [7]. The most evident alterations in gait, besides a marked asymmetry, include walking speed reduction, longer durations of double-stance and paretic swing phases, reduced paretic single-stance phase duration, cadence, and stride length [3,8], asymmetric postural behavior during walking and standing [9], altered kinematics, and reduced ankle push-off ability in terminal stance [10,11]. In this context, gait impairments cause difficulties with performing the activities of daily living and mobility, thus reducing independence and quality of life [12]. The different degrees of impairment that characterize the affected and not-affected side suggest that the study of gait symmetry represents a crucial feature in characterizing and quantifying locomotion in hemiparetic individuals, also considering that an asymmetric gait pattern is generally characterized by poor efficiency and requires high energy expenditure. In addition, the restoration of gait symmetry is not only an indicator of functional recovery but also an important aim for clinical rehabilitation practice.

Generally, the gait asymmetry metrics of both healthy [13] and injured individuals [14,15,16] are based on the assessments of right vs. left spatio-temporal parameters, kinematics (e.g., joint angles), and kinetics (e.g., ground reaction forces, GRFs) carried out using three-dimensional motion analysis systems. However, due to a number of issues associated with high cost, operator skills, and space requirements, their application is often limited to research settings [17]. Therefore, it appears important to have available reliable quantitative tools that are suitable for clinical daily use and are able to effectively quantify gait asymmetry. For this purpose, wearable accelerometers represent a very appealing option due to their relatively low cost and ease of use, and are becoming widespread in investigating several aspects of human movement in a variety of contexts [18]. Usually, gait asymmetry is quantified on the basis of conventional spatio-temporal parameters, including step time asymmetry, stance time asymmetry, swing time asymmetry, and step length asymmetry; these are calculated as the absolute difference between consecutive left and right steps. However, further asymmetry variables derived from the cyclical acceleration signals during gait have been found to be effective in detecting gait alterations. In particular, trunk accelerations acquired during gait, using a single sensor placed on the lower back, allows us to obtain information about the so-called “smoothness” of gait (also defined as step-to-step symmetry) by means of a parameter called the Harmonic Ratio (HR) [19,20,21]. The HR is based on a spectral analysis of the acceleration signals and is related to the bilateral rhythmicity of movement, based on the measure of trunk acceleration during a stride that is expected to be formed by two alternating symmetric steps; it provides different kinds of information with respect to the traditional spatio-temporal parameters, which are focused on the lower limb symmetry at the distal level [22]. Recent studies demonstrated that the HR parameter is worthwhile in quantifying gait alterations associated with neurologic and orthopedic conditions, such as older people [23], Parkinson’s disease patients [22], multiple sclerosis [24], normal weight and obese children/adolescents [25,26], Prader–Willi patients [27,28], and cognitively impaired individuals [29]; in several cases, it is able to reveal subtle changes in gait that might occur well before they become detectable in terms of conventional spatio-temporal parameters [22,24,25,26,30]. Furthermore, it must be emphasized that trunk accelerations could be easily recorded by a single sensor in clinical settings or in other ecological contexts, without the limitations of a movement analysis laboratory, which requires expensive equipment, long setup times, and time-consuming post-processing procedures [26,28,30,31].

Concerning its application on individuals with stroke, to date, several studies reported that the HR could be considered a robust outcome in quantifying the step-to-step asymmetry during gait [18,32,33,34,35,36,37]. However, to the best of our knowledge, the HR has not been used so far as an indicator of the effectiveness of rehabilitative treatment targeting the improvement of gait in individuals with stroke. Thus, in the present study, we employed the HR to quantify the possible changes originating from the use of the foot drop stimulator (FDS) on gait asymmetry in chronic post-stroke subjects while walking in an outdoor environment. FDSs are based on the functional electrical stimulation (FES) of the peroneal nerve to elicit ankle dorsiflexion during the swing phase of the step cycle [38,39,40]. Such devices have been proven to be effective in enhancing gait speed in short- and long-term studies [41,42,43], but there is no evidence about the reduction of step-to-step symmetry.

The FDS device seems to have positive effects on gait in stroke patients [40,44]; thus, it appears of interest to assess the feasibility of the HR and to compare it to conventional spatio-temporal measures. Our hypothesis is that changes in the spatio-temporal parameters of gait previously reported [41,43] could also be accompanied by modifications of step-to-step symmetry in stroke patients.

## 2. Materials and Methods

### 2.1. Study Design

This quasi-experimental clinical trial was registered at ClinicalTrial.gov (NCT04266899) and approved by the Ethics and Research Committee of the Santa Casa de Misericórdia Hospital of Porto Alegre (CAAE 64819617.0.0000.5335). Procedures were conducted according to the Template for Intervention Description and Replication (TIDeR) checklist [45].

### 2.2. Participants

Participants were recruited through a database of the Santa Casa de Misericórdia Neurology service in Porto Alegre and social networks, and then selected according to eligibility criteria. We included individuals with ischemic or hemorrhagic chronic stroke confirmed by head Computerized Tomography (CT) or Magnetic Resonance Imaging (MRI) at least 6 months before recruitment, aged 20 to 80 years, with mild (29–34/34), moderate (20–28/34), or severe hemiparesis (0–19/34) according to the Fugl–Meyer score’s lower limb subdivision [46]. Patients had to have minimal cognitive ability on the Mini-Mental State Examination (>20 points (illiterate) or >24 (literate)) [47], and no history of seizures or recent episodes of a fall (at least 3 months before study engagement). In addition, participants were required to be able to walk at least 30 m autonomously without assistive devices. Individuals who presented any contraindication for electrical stimulation were excluded (any electric or metallic implant; skin problems or lesion in proximity to the site of FDS stimulation; pregnancy). Furthermore, subjects with any lower limb musculoskeletal disorder, significant visual impairment, low response to FDS electrical stimulation (no response to the highest stimulation intensity provided by the FDS device, namely 200 mA intensity), or relevant ankle restriction (fixed ankle contracture at ≥10 degrees of plantar flexion in the hemiplegic leg with the knee extended) were also excluded.

A group of healthy individuals (Control Group: CG) matched by age and sex were also tested. Exclusion criteria for the CG were the existence of cardiorespiratory, neurological, or musculoskeletal disorders. All of them exhibited normal flexibility and muscle strength, had no evident gait abnormalities, and were able to walk independently.

The experimental protocol was carried out in accordance with the ethical standards of the institute and the 1964 Declaration of Helsinki and its later amendments. All participants signed a free and informed consent form before enrolment.

### 2.3. Procedures

This study was conducted at the Movement Analysis and Rehabilitation Laboratory of the Federal University of Health Sciences of Porto Alegre (UFCSPA) between January 2018 and May 2019. Each participant participated in a clinical and documented evaluation session. Indirect assessment of spasticity was done by the Modified Ashworth Scale (MAS) [48], which consists of five ordinal values ranging from 0 (no tonus increase) to 4 (stiffness) [48]. Participants were evaluated while lying in a supine position and were instructed to remain relaxed during the test. The spasticity of plantar flexors, knee extensors, and hip adductors was tested. All clinical assessments were performed by the same researcher at baseline (pre-intervention; 2 days before the first session) and post-intervention (1 day after the last session).

### 2.4. Intervention

**Figure 1:**
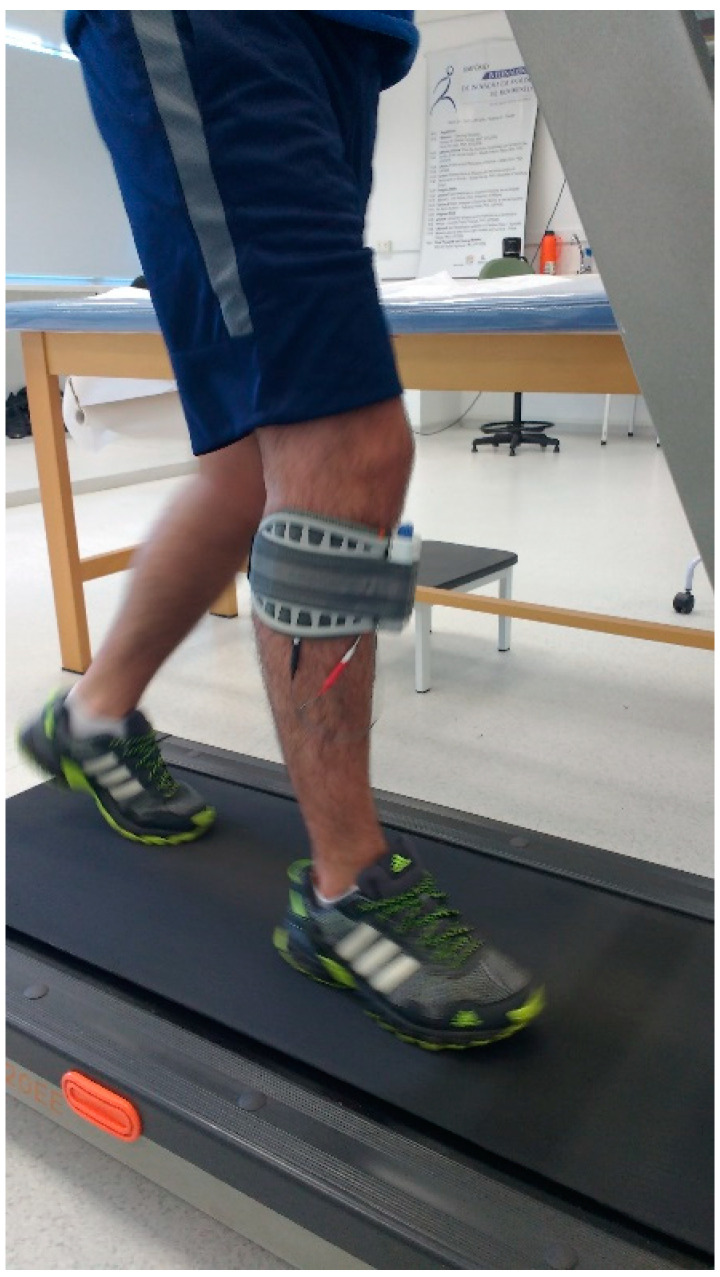
The experimental setup.

Subjects underwent 10 face-to-face sessions of 20 min of walking on a treadmill (Athletic advanced 720EE, Buenos Aires, Argentina) with a self-selected comfortable velocity while using the FDS device, configured according to each subject’s need (Figure 1).

The WalkAide device (Innovative Neurotronics, Austin, TX, USA) was used to stimulate the peroneal nerve on the affected side through a tilt sensor that detects the affected leg tilt when the foot contact on the ground changes from posterior to anterior (pre-swing phase). The stimulus stops when the leg is tilted forward on foot strike [49,50]. Before treatment, subjects underwent a 3-day adaptation period with the FDS device that included walking overground on a flat surface, walking up and down stairs, and walking on a treadmill. The FDS device was set at enough intensity to achieve the movement, but, at the same time, was required to be comfortable. Before each session, subjects underwent 15 min of lower limb stretching and vital sign measurements. The training sessions, which were interspersed by 5 min rest periods, consisted of 20 min of walking on a treadmill. Each session was administered by the same physical therapist that annotated the overall covered distance and also periodically checked subjects’ heart rate and blood pressure. Subjects were allowed to stop the trial at any time, if necessary.

### 2.5. Data Acquisition

A single miniaturized inertial sensor (G-Sensor^®^, BTS Bioengineering, Milano, Italy), previously validated for investigations on gait in unaffected individuals and people with neurologic conditions [25,26,27,51,52,53], was placed on the participants’ lower back, approximately at the L4–L5 vertebrae position. The sensor, which is sized 70 mm × 40 mm × 18 mm and weighs 37 g, is composed of a three-axis accelerometer, a three-axis gyroscope, and a three-axis magnetometer. After a brief familiarization phase, participants were asked to walk at a self-selected speed on a 30-m flat pathway of the university outdoors. The gait test was performed before and a day after the 10-session treatment and named as PRE and POST session, respectively. Each trial was repeated three times and a mean of them was calculated. Participants were equipped with the FDS device during the gait assessment of the PRE and POST session.

The values of the linear accelerations along the antero-posterior (AP), medio-lateral (ML), and vertical (V) directions were acquired by means of the inertial sensor at a frequency of 100 Hz. The elaboration and parameter computations were performed with a custom Matlab^®^ routine. The first 5 s of the acquisition (during which the subject was requested to stand still) were used to verify the orientation of the sensor, and this information was then used to correct the acceleration vectors’ data during the gait trial.

Based on the raw acceleration data, the main spatio-temporal parameters (gait speed, stride length, cadence, and duration of stance and double support phase) were calculated following the approaches described in the literature [27,54,55]. The HRs for the AP, ML, and V directions were computed according to the procedure proposed by Pasciuto et al. [20].

### 2.6. Statistical Analysis

Sample size was determined by G-Power 3.0 software (version 3.1.9.4.; Faul & Buchner, Germany) based on a previous study [18] considering the minimum effect size of 0.56% to detect a minimum clinical difference in the HR of the ML direction. Sample size was calculated by adopting 90% power and an alpha value of 0.05. A total of 29 participants was calculated as necessary to perform this study. The parametric Student’s t-test, non-parametric Mann–Whitney U test, and Chi-square test were used to compare the demographic characteristics between the stroke and the control groups. After verifying their normality (using the Shapiro–Wilk test) and homogeneity of variances (Levene’s test), a one-way analysis of variance for repeated measure (RM-ANOVA) was conducted using SPSS software (v.20, IBM, Armonk, NY, USA) to verify the effect of the use of the foot drop stimulator (FDS) on spatio-temporal parameters and the HR PRE and POST training. Time (PRE/POST) was set as an independent variable, while the five gait parameters previously listed and the three HRs represented the dependent variables. After the Bonferroni correction was performed considering the three main outcomes (HR in the AP, ML, and V direction), the statistical significance was fixed at *p* = 0.017. The Student’s t-test assessed the differences between PRE evaluations and the controls (Control Group).

## 3. Results

Participants were recruited from March 2017 to August 2019, while the final measurements were carried out in August 2019. Forty-one stroke survivors were contacted and the final tested sample included 32 individuals. Their baseline demographic and clinical characteristics are reported in Table 1.

Table 2 presents the values of the spatio-temporal parameters and the HR features for stroke patients in the PRE and POST session and for the Control Group. In the comparison of the PRE session of stroke individuals vs. the Control Group, all parameters exhibited significant differences, with the exception of the double support phase.

As for the assessment of time effects (PRE vs. POST session in stroke patients), a significant effect of time was found and, in particular, the post-hoc analysis detected a significant increase in gait speed (*p* = 0.028). Regarding the symmetry parameters (HR features), the statistical analysis detected a significant effect of time for the HR in all three directions. In particular, the HR in the ML direction increased (+20%, *p* = 0.02), while those in the AP and V directions decreased after training (both approximately 13%, AP direction: *p* = 0.003, V direction: *p* = 0.007).

## 4. Discussion

The purpose of the present study was to assess the effects of FDS use in individuals with stroke by means of spatio-temporal and step-to-step symmetry parameters obtained from trunk accelerations acquired using a wearable inertial sensor.

In terms of the spatio-temporal parameters, the only significant change observed post-treatment involved gait speed. This result is consistent with previous, similar studies [41,42,43]. However, it is worth noting that such improvement (+0.04 m/s), although statistically significant, was lower than the value indicated in the literature as clinically meaningful; several authors reported that the minimally clinical important difference in individuals who undergo inpatient rehabilitation after stroke lies between 0.10 and 0.18 m/s [56,57]. Concerning the HR parameters, mixed effects were observed in the treatment. Generally speaking, higher HR values denote better gait stability [30] and improved symmetry, smoothness, and rhythmicity. Our results show that the HR in the ML direction significantly increased, and this result may be explained by the fact that, during gait, the central nervous system controls the ML displacements related to the weight acceptance of each step [58,59]. However, in hemiplegic subjects the lack of lower muscle strength and increased instability observed in the affected side often acts by disrupting this strategy [60,61,62,63,64,65,66,67]. In this sense, the FDS walking training may have increased the ML gait symmetry by generating better foot contact [68] while shifting the body weight to a medial position, resulting in an improved ML stability during the walking movement and possibly, in the medium term, improving muscle strength and spasticity [69]. The important role played by the HR in the ML direction as a determinant of stability is also confirmed by previous studies reporting that a good lateral harmonic stability in gait may be important for minimizing fall risk in older people [70,71]. In addition, however, the *p* value observed in the HR of the ML direction is slightly higher than the post-Bonferroni correction fixed statistical significance; the improvement of the HR in the ML direction is highly clinically relevant (+20%) and must be taken into consideration. Furthermore, it is important to note that the improvement of the HR appears in the more critical direction, the ML direction, which exhibited, in the PRE session, a much lower value than the control group value with respect to the V and AP directions.

In contrast, we also detected a reduction of the AP and V components of the HR, even though it is worth noting that the magnitude of such changes is approximately half compared with those related to the ML direction (−13% vs. +20%). This result suggests that the number of training sessions may be insufficient to let participants completely adapt to the new gait strategy. Further studies are thus necessary to verify whether a longer training period may trigger a complete readaptation of gait, from the point of view of symmetry, which involves all directions. This hypothesis is somewhat supported by previous studies that report how neuroprostheses are effective in enhancing balance control during walking (and thus effectively manage foot drop) after 8 weeks [41].

In addition, it is important to consider that our study’s participants are mainly severely impaired. In this view, they probably need longer intervention periods to exhibit substantial changes in walking symmetry, which may contribute to the lack of improvement in the AP and V components of the HR.

While walking, maintaining balance requires continuous integrative control, especially in the ML direction, in order to cope with instability during single limb support [72]; thus, we can hypothesize that, after the treatment, patients are trying to adapt to the new gait strategy, which is characterized by higher velocity too, and the importance is given to the ML direction. Furthermore, it has been demonstrated that the HR is speed-dependent and it is especially affected in the very slow condition [73]. It is important to underline that, in this study, only the HR and spatio-temporal parameters were investigated; further research should be conducted, integrating these parameters with kinematic and kinetic data, to evaluate the most sensitive measures to changes in walking due to the FDS. Kinematics and kinetics may clarify where the symmetry deviations occur. In addition, in this study, no placebo control group was included. Further research that includes a placebo control group should be conducted in order to distinguish, more clearly, whether the procedure is effective or if the changes could be associated with a placebo effect.

Future studies may also wish to assess the effects of the FDS at a longer follow-up to more fully understand if the gait changes are maintained over time. In addition, this study contains a disproportionate number of men, who make up 68.8% of the study participants. Thus, by increasing the number of female patients, further work could be conducted to better understand the possible sex-related differences in trunk movement asymmetry. In addition, a large range of time since stroke (from 6 to 96 months) could have influenced the results. Further studies with a larger sample size and with a restricted range of time since stroke should investigate whether the results differ in stroke patients with dissimilar levels of impairment (i.e., mild–moderate vs. severe impairment).

Even though this study presents some limitations, it presents two original aspects: (1) the assessments were conducted using an inertial wearable sensor to document the effects of the FDS on gait in stroke patients; and (2) the analysis was conducted considering not only the traditional spatio-temporal parameters but also the HR, which has never been used to quantify the modifications of step-to-step symmetry in stroke patients induced by FDS treatment.

## Figures and Tables

**Table 1:** Participant characteristics.

	Stroke	Control Group	*p* Value
	(*n* = 32)	(*n* = 32)	
Gender, *n* (%)			
Male	22 (68.8)	22 (68.8)	0.444 ^¥^
Age (years)	56.84 ± 9.10	56.81 ± 8.88	0.989 ^#^
Height (m)	1.68 ± 9.73	1.72 ± 8.23	0.221 ^#^
Body mass (kg)	75.13 ±11.90	78.26 ± 12.97	0.318 ^*^
Time since stroke (months) (min–max)	39.41 (6–96)		
Stroke type, *n* (%)			
Ischemic	24 (75)		
Hemorrhagic	8 (25)		
Affected hemisphere, *n* (%)			
Right	19 (59.4)		
Left	13 (40.6)		
FMA–LL (0–34), (min–max)	19.63 (11–32)		
MAS, frequency (0/1/1 + /2/3/4)			
Plantiflexors	0/3/2/4/11/12		
Knee extensors	5/7/6/4/8/2		
Adductors	5/4/4/12/7/0		

FMA—LL, Fugl–Meyer Assessment—Lower Limb; MAS, Modified Ashworth Scale; max, maximum; min, minimum; *n*, number of participants; SD, standard deviation. ^#^ *p*-value parametric Student’s t-tests; ^*^ *p*-value nonparametric Mann–Whitney and ^¥^ *p*-value Chi-square tests were used to compare the demographic characteristics between the stroke and control groups.

**Table 2:** The spatio-temporal parameters of gait and the Harmonic Ratio values of the participants.

	Stroke				Control Group	
	PRE	POST	F	*p* Value		*p* Value
**Spatio-temporal parameters**						
Gait speed (m/s)	0.62 ± 0.47	0.66 ± 0.25 ^*^	4.615	0.040	1.22 ± 0.23	<0.001 ^#^
Stride length (m)	1.28 ± 0.47	1.23 ± 0.48	3.23	0.082	1.47± 0.12	0.044 ^#^
Cadence (steps/min)	85.06 ± 26.78	88.64 ± 25.81	4.49	0.043	116.99 ± 9.60	<0.001 ^#^
Stance phase (% Gait Cycle)	55.66 ± 7.98	56.17 ± 6.92	0.845	0.365	59.46 ± 1.40	0.010 ^#^
Double support phase (% Gait Cycle)	9.72 ± 4.26	10.13± 5.03	0.80	0.379	9.82 ± 1.50	0.102
**Harmonic Ratio**						
AP direction	80.87 ± 11.24	71.44 ± 18.00 ^*^	10.47	0.003	95.12 ± 2.33	<0.001 ^#^
ML direction	38.02 ± 19.92	47.65 ± 21.44 ^*^	6.05	0.020	85.61 ± 8.03	<0.001 ^#^
V direction	72.38 ± 11.89	64.13 ± 19.97 ^*^	8.39	0.007	95.18 ± 2.02	<0.001 ^#^

Values are expressed as mean ± SD. PRE = pre-training (after habituation with FDS—3 days); POST = post-training (after 10 sessions of intensive training with the Foot Drop Stimulator); AP = Antero-posterior; ML = medio-lateral; V = vertical; ^*^ significant difference between PRE and POST training: One-way Repeated Measure Analysis of variance (spatio-temporal parameters *p* = 0.05; Harmonic Ratio parameters’ statistical significance after Bonferroni correction (*p* < 0.017); ^#^ significant difference when comparing the PRE intervention and control group: Student’s t-test (*p* < 0.05).

## Data Availability

The data presented in this study are available on request from the corresponding author.
